# Efficient CRISPR/Cas9-Mediated Gene Editing in *Arabidopsis thaliana* and Inheritance of Modified Genes in the T2 and T3 Generations

**DOI:** 10.1371/journal.pone.0099225

**Published:** 2014-06-11

**Authors:** WenZhi Jiang, Bing Yang, Donald P. Weeks

**Affiliations:** 1 Department of Biochemistry, University of Nebraska, Lincoln, Nebraska, United States of America; 2 Department of Genetics, Development and Cell Biology, Iowa State University, Ames, Iowa, United States of America; Leibniz-Institute for Vegetable and Ornamental Plants, Germany

## Abstract

The newly developed CRISPR/Cas9 system for targeted gene knockout or editing has recently been shown to function in plants in both transient expression systems as well as in primary T1 transgenic plants. However, stable transmission of genes modified by the Cas9/single guide RNA (sgRNA) system to the T2 generation and beyond has not been demonstrated. Here we provide extensive data demonstrating the efficiency of Cas9/sgRNA in causing modification of a chromosomally integrated target reporter gene during early development of transgenic Arabidopsis plants and inheritance of the modified gene in T2 and T3 progeny. Efficient conversion of a nonfunctional, out-of-frame *GFP* gene to a functional *GFP* gene was confirmed in T1 plants by the observation of green fluorescent signals in leaf tissues as well as the presence of mutagenized DNA sequences at the sgRNA target site within the *GFP* gene. All GFP-positive T1 transgenic plants and nearly all GFP-negative plants examined contained mutagenized *GFP* genes. Analyses of 42 individual T2 generation plants derived from 6 different T1 progenitor plants showed that 50% of T2 plants inherited a single T-DNA insert. The efficiency of the Cas9/sgRNA system and stable inheritance of edited genes point to the promise of this system for facile editing of plant genes.

## Introduction

In recent years, zinc finger nuclease (ZFN) technology [1] and TAL Effector Nuclease (TALEN) technology [2]–[5] have become powerful gene editing tools for targeted gene modification in human cells, fruit flies, zebrafish, nematodes and plants. For both ZFNs and TALENs, engineered sequence-specific DNA binding domains are fused with a subunit of the nonspecific DNA nuclease, Fok1. As a result, pairs of ZFNs and TALENs targeting adjacent DNA target sites generate double strand breaks (DSBs) at or near the target site. Repair of the DSB by error-prone nonhomologous end joining (NHEJ) or homologous recombination (HR) often lead to gene sequence modification, including gene knockout. Within the past year, another highly promising system for gene editing, the clustered regulatory interspersed short palindromic repeat (CRISPR)/CRISPR-associated protein (Cas) system, has evolved from studies of bacterial defense systems that provide protection against invading viruses or plasmid DNAs [6]–[8]. CRISPR loci are variable short spacers separated by short repeats and are transcribed and processed into short non-coding RNAs. These short RNAs can form a functional complex with Cas proteins and guide the complex to cleave complementary foreign DNAs. The type II CRISPR/Cas system derived from *Streptococcus pyogenes* is the most widely used for gene editing [6]–[9]. It has the marked advantage of possessing a required PAM recognition sequence of only two nucleotides (GG). Development of single guide RNAs (sgRNAs) that are fusions of essential portions of tracrRNA with the “guide RNA” of crRNAs was an important improvement in facilitating rapid adoption of the CRISPR technology for targeted gene modification in eukaryotic cells [6],[9]. To obtain a functional RNA-guided gene disruption in a host cell, one needs only to transform the cell with the *Cas9* gene and a gene (generally driven by an RNA polymerase III-dependent promoter) encoding a sgRNA that contains a 20 bp sequence complementary to the segment of DNA in the host cell that is the target for disruption by a DSB. Once a host cell containing the *Cas9* gene has been established, subsequent modification of a target gene requires only transformation with a sgRNA gene producing a sgRNA complementary to the target gene. Simultaneous modification of two or more genes simply requires transformation of cells with two or more appropriately targeted sgRNA genes [e.g., 10]. The Cas9/sgRNA system has been used successfully for gene disruption, gene activation/repression and various other kinds of genome editing in several types of cells and organisms [e.g., 8–19] – with a number of other gene editing functions possible in the near future [20].

Very recently, there were reports of successful expression of the CRISPR/Cas9 system in higher plant tissue culture systems and during transient expression in *Agrobacterium* inoculated plant cells and tissues [16],[21]–[26]. In this report we demonstrate that the Cas9/sgRNA system delivered by *Agrobacterium tumefaciens* is fully functional when delivered to Arabidopsis by the floral dip method. Results using T1 transgenic Arabidopsis are presented showing efficient targeting of specific DNA sequences for DNA cleavage and error-prone repair by NHEJ (i.e., successful conversion of an out-of-frame mutant *GFP* gene to a functional *GFP* gene that provides a visual demonstration and verification of Cas9/sgRNA activity). Results with progeny from several independent T1 plants have been used to demonstrate successful inheritance of modified genes in the T2 and T3 generations and to suggest potential silencing of the Cas9/sgRNA beyond the T1 generation in Arabidopsis.

## Results And Discussion

### Strategy For Detection Of Cas9 And Sgrna Induced Mutagenesis In Arabidopsis

**Figure 1:**
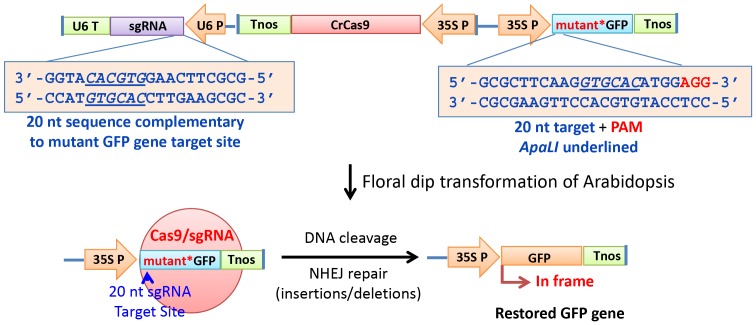
Design of a Cas9/sgRNA system for mutagenesis and restoration of activity of a non-functional (out-of-frame) mutant *GFP* gene in Arabidopsis.

Double strand DNA breaks (DSBs) at the target site in a non-functional *GFP* gene caused by ZFN, TALEN and Cas9/sgRNA expression are most often repaired by the non-homologous end joining (NHEJ) DNA repair mechanism. This repair is often accompanied by small deletions or insertions of nucleotides at the site of repair. We previously took advantage of this mechanism to develop a reporter system for detecting transient expression of Cas9/sgRNA activity in Arabidopsis leaves transformed with two *Agrobacterium* lines, one carrying binary vectors containing the Cas9 and sgRNA genes and another line carrying a nonfunctional, out-of-frame, *GFP* gene [16]. For present studies aimed at generating stably transformed Arabidopsis plants expressing the Cas9/sgRNA genes, we used the same GFP reporter system (outlined in Figure 1) that places all three required genes on a single binary vector instead of the two vectors previously employed. This reporter system involves use of a nonfunctional mutant version of a *GFP* gene containing a small 20 nucleotide insertion (plus a 3 nucleotide extension that includes a GG PAM recognition sequence) immediately downstream of the ATG start codon of the *GFP* gene. This insert was designed to create a shift in the reading frame of the gene so the products lack the ability to produce a fluorescent signal in transgenic plant cells. A single guide RNA (sgRNA) gene construct containing a 20 bp sequence complementary to the 20 nucleotide target in the mutant GFP gene was engineered to be driven by the Arabidopsis U6 gene promoter. Incorporation of the sgRNA produced from this gene into the Cas9 protein should guide the Cas9/sgRNA complex to the 20 bp target sequence in the mutant *GFP* gene and cause subsequent double strand cleavage of the *GFP* gene target sequence. NHEJ DNA repair of the DSB involving insertion or deletion of nucleotides at the cleavage site would, in approximately one-third of the cases, reestablish a correct reading for the *GFP* gene. In such cases, the mutation should allow production and visualization of green fluorescent protein in transgenic plant cells. Important advantages of this system are that it generates a positive GFP signal that is easily detected in even small clusters of plant cells and that expression of GFP has no negative influence on cell or plant physiology - as is the case for a number Cas9/sgRNA targets used in previous studies with plants or plant cells [21]–[26]. In addition to our previous success in using restoration of GFP signals from a mutant GFP gene in Arabidopsis and tobacco leaf tissues transiently transformed with Cas9/sgRNA genes [16], others [22] have successfully used a similar reporter system (a nonfunctional split YFP target gene) to obtain Cas9/sgRNA-dependent transient expression of YFP in Arabidopsis protoplast cells and detection of the positive signal via flow cytometry. Unlike earlier studies with plant cells and tissues [21]–[26], we sought both to gauge the efficiency with which Cas9/sgRNA can mutagenize a target gene and to demonstrate that such a gene can be stably inherited in second and third generation transgenic plants.

**Figure 2:**
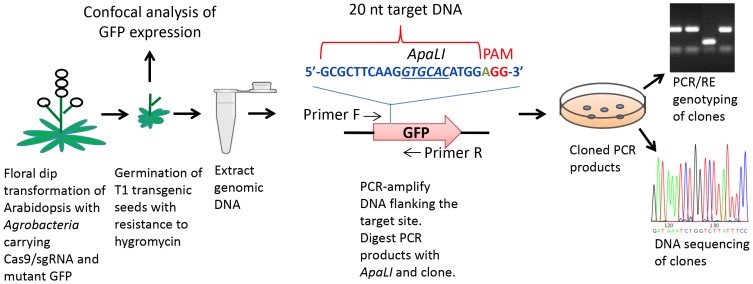
Cas9/sgRNA-mediated mutagenesis in Arabidopsis. Scheme for Cas9/sgRNA-mediated mutagenesis of a non-functional (out-of-frame) mutant *GFP* gene, detection of T1 Arabidopsis leaves with restored *GFP* gene function by confocal microscopy and documentation of target site DNA mutagenesis.

As outlined in Figure 2, Arabidopsis plants were transformed with *Agrobacteria* using a floral dip method [27]. The treated plants were allowed to develop and resulting T1 generation seeds were germinated on plates containing solid MS medium and hygromycin. First or second true leaves of hygromycin resistant T1 seedlings were analyzed by confocal microscopy for the presence or absence of green fluorescent signals. PCR primers flanking the *GFP* gene target site were used to amplify the target region. The amplified target regions were genotyped by a PCR/restriction enzyme digestion protocol that allowed detection of mutagenized sgRNA target sites by loss of an *Apa*LI restriction site. The *Apa*LI resistant fragments were cloned and mutagenesis of the sgRNA target sites in several T1 Arabidopsis plants was verified using DNA sequencing of the target sequence. Finally, T2 and T3 progeny of several T1 plants were analyzed to confirm inheritance of mutagenized *GFP* genes and to assess the presence or absence of Cas9/sgRNA activity in the T2 and T3 generation.

### Evidence For High-Level Expression Of Cas9 And Sgrna Genes In T1 Arabidopsis Plants

**Figure 3:**
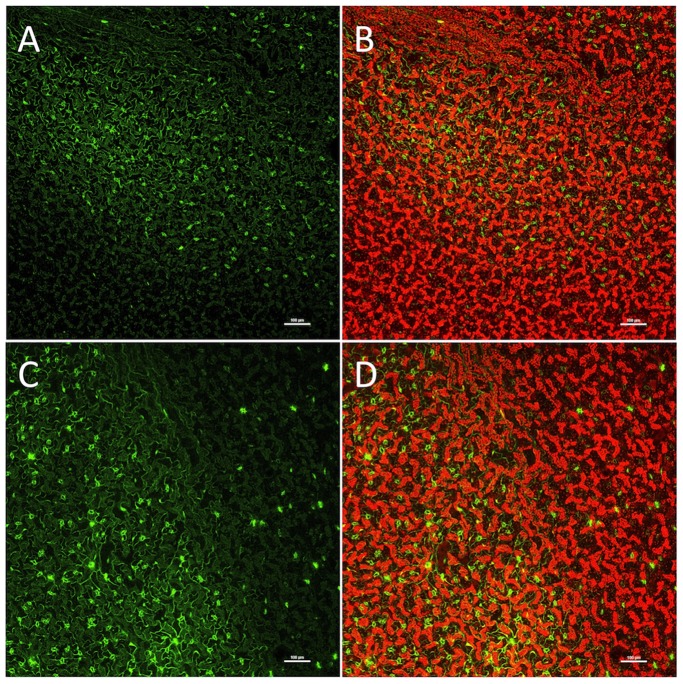
Cas9/sgRNA-mediated mutagenesis of nonfunctional mutant *GFP* genes in Arabidopsis leaves. Expression in T1 Arabidopsis leaf cells of functional *GFP* genes produced by Cas9/sgRNA-mediated mutagenesis of nonfunctional mutant *GFP* genes introduced along with the Cas9 and sgRNA genes using *A. tumefaciens* and a floral dip transformation protocol. A and C) Detection of green fluorescence protein signals in transgenic leaves. B and D) Merged image of red chlorophyll fluorescence and GFP fluorescence. Leaves from hygromycin resistant seedlings were photographed ten days (A and B) and twenty days (C and D) after seed germination. Bar, 100 µm.

Of the seeds collected from T0 parental plants approximately 1% germinated in the presence of hygromycin. This typical recovery rate for transformants using the floral dip method indicated no marked negative effect of the Cas9/sgRNA system on the efficiency of Arabidopsis transformation or seed development. Examination by confocal fluorescence microscopy of leaves of resulting hygromycin-resistant TI plants showed widespread presence of functional *GFP* genes in leaf cells (Figure 3). Such fluorescence was observed with leaves of 35 of 60 T1 Arabidopsis plants obtained by germinating T1 seeds on plates containing levels of hygromycin (20 µg mL^−1^) lethal to germinating seedlings of wild type plants. As a negative control, Arabidopsis inflorescences were inoculated with *Agrobacteria* containing a binary vector identical to the one described above but carrying a sgRNA gene producing a randomly selected 20 nucleotide targeting sequence plus an AGG PAM sequence (GGATAACATGGCCATCATCAAGG) that was not complementary to the sgRNA target site in the nonfunctional *GFP* gene or to any Arabidopsis genome sequence. None of the 61 hygromycin resistant T1 plants obtained in this experiment displayed green fluorescence in their leaf cells or other plant parts (data not shown). These results demonstrated that neither the floral dip transformation procedure nor the Cas9 gene alone, per se, were responsible for mutagenesis of the out-of-frame *GFP* gene (i.e., successful mutagenesis required a sgRNA gene encoding a properly targeted sgRNA sequence as well as the Cas9 gene).

Two notes should be made in regard to our choice of the conversion of a nonfunctional GFP gene to an active GFP as an indication of Cas9/sgRNA activity. First, the visual signals created in leaf tissues was not meant to be used as an accurate measure of mutagenesis rates – rather we have relied on more accurate measures offered by restriction enzyme analyses of PCR products and DNA sequencing (described below). Second, we wished to determine when during seed and plant development the Cas9/sgRNA complex was active in causing targeted gene disruptions. The later aim was accomplished when it was observed that in essentially all of the transgenic plants expressing GFP, there was a patchy pattern of GFP expression seen across the surface of individual leaves (Figure 3A and 3C). The patchiness of GFP expression contrasts sharply with the uniform distribution of chlorophyll fluorescence captured in the same photographic frames (Figure 3 B and 3D). The simplest explanation for the mosaic patterns observed in plants expressing the Cas9 and sgRNA genes is that while the T-DNA region carrying the Cas9, sgRNA and nonfunctional *GFP* genes is inserted into the chromosome of an ovule progenitor cell prior to fertilization [28], the action of the Cas9 and sgRNA complex in binding and mutagenizing the nonfunctional *GFP* gene and converting it to a functional *GFP* gene is a stochastic process that can occur in somatic cells anytime during seed development and, perhaps, leaf development. The presence in a single leaf of large numbers of different mutagenized DNA sequences in the target region of the *GFP* gene (along with nonmutagenized sequences) (documented and discussed below) strongly suggests that this explanation is correct.

**Figure 4:**
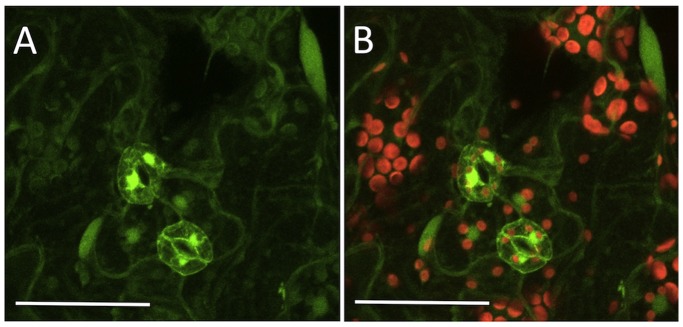
Robust expression in guard cells of functional *GFP* genes created by Cas9/sgRNA-mediated mutagenesis. A) Detection of green fluorescence protein signals in guard cells of transgenic leaf stomata with lesser expression in surrounding leaf epidermal cells of T1 transgenic Arabidopsis plants. B) Merged image of red chlorophyll fluorescence and GFP fluorescence. Bar, 50 µm. Photographed twenty days after seed germination.

Another form of variation in expression of GFP across the surface of the leaf was also evident when comparing green fluorescence intensities emanating from stomatal guard cells with surrounding epidermal cells (Figure 4). This differential expression pattern was observed consistently among all the GFP-positive leaves examined and is similar to the preferential expression of *GFP* genes driven by the 35S CaMV promoter in guard cells relative to expression in surrounding cells observed by others [29].

### Analysis Of Relative Abundance Of Mutagenized And Nonmutagenized *Gfp* Genes In Leaves Of T1 Arabidopsis Plants

**Figure 5:**
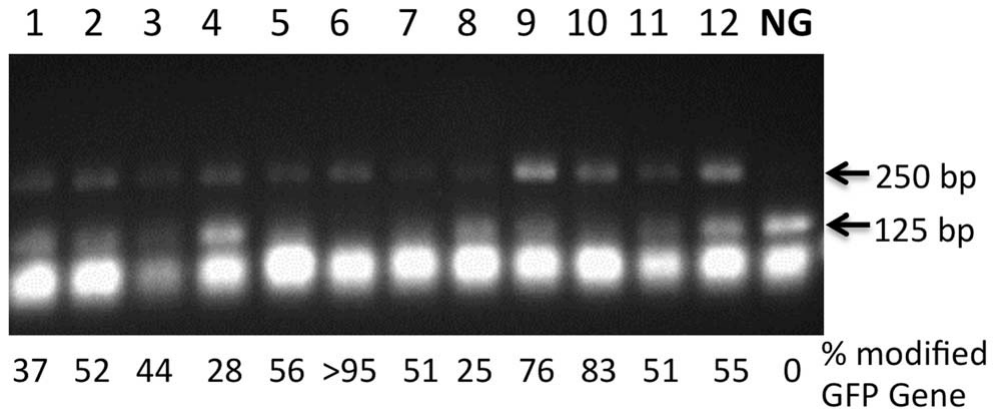
Efficiency of Cas9/sgRNA mutagenesis in Arabidopsis plants. PCR/Restriction Enzyme (PCR/RE) analysis of total DNA extracts from individual hygromycin resistant T1 Arabidopsis plants (lanes 1–12) showing the relative proportion of nonfunctional *GFP* genes mutagenized by Cas9/sgRNA activity. Bottom arrow indicates the expected ∼125 bp DNA fragments resulting from *Apa*LI cleavage of the ∼250 bp PCR product amplified from nonfunctional, out-of-frame, nonmutagenized *GFP* genes that contains an intact *Apa*LI cleavage site in the sgRNA target region. Top arrow indicates the expected ∼250 bp size of PCR products from *GFP* genes mutagenized by the Cas9/sgRNA system in such a manner that they are no longer are susceptible to cleavage by *Apa*LI. NG (Nonfunctional *GFP* Gene), the PCR products amplified from a sgRNA target site of a cloned nonfunctional, out-of-frame, *GFP* gene digested with *Apa*LI. % modified *GFP* Gene = (pixels in 250 bp band)/(pixels in 250 bp band+pixels in 125 bp band) ×100.

A combination of PCR amplification of the sgRNA target region within the mutant *GFP* gene and restriction digestion using the enzyme *Apa*LI was used to obtain a rough estimate of the efficiency of Cas9/sgRNA-mediated mutagenesis. The aim of this analysis was to determine the proportion of nonfunctional, out-of-frame, *GFP* genes that were not mutagenized by the Cas9/sgRNA system in T1 plant cells relative to the proportion of genes in which the Cas9/sgRNA complex was successful in altering the *Apa*LI recognition site within the nonfunctional *GFP* gene. To accomplish this, total DNA was extracted from a single leaf of 12 different hygromycin-resistant, GFP-positive, T1 Arabidopsis seedlings. These DNA samples were subjected to PCR amplification with DNA primers complementary to sites approximately 125 bp on either side of the *Apa*LI cut site within the 20 bp sgRNA target site of the mutant *GFP* gene (Figure 2). Digestion of the approximately 250 bp PCR product from amplification of a cloned nonfuctional, out-of-frame, *GFP* gene with *Apa*LI resulted in the expected production of two DNA fragments each of approximately 125 bp in length (Figure 5, lane NG). The *Apa*LI digestion patterns of PCR-amplified DNA from the 12 individual T1 plants (all displaying GFP positive leaf segments) revealed that all (as expected) contained a 250 bp, *Apa*LI-resistant DNA product and that the portion of target DNA sequences mutagenized by the Cas9/sgRNA complex ranged from 37% to greater than 95% (Figure 5, lanes 1–12). These data demonstrate a high level of Cas9/sgRNA-driven DNA cleavage and resulting gene mutagenesis in somatic cells of Arabidopsis T1 plants.

As an extension of the experiments described above, we also examined 12 out of 25 GFP-negative T1 Arabidopsis plants to determine if they contained mutagenized target *GFP* genes as ascertained by PCR/restriction enzyme analysis (Figure S1) similar to those depicted in Figure 5. Interestingly, 11 out of 12 GFP-negative plants examined were shown to have at least one mutagenized target site (data not shown). This again demonstrated a high frequency of Cas9/sgRNA-induced mutagenesis. The large proportion of GFP negative T1 plants (11 out of 12) containing at least one mutagenized target region illustrates, as expected, that only a fraction (∼one-third) of mutagenized target sites restore a proper reading frame to the coding region of the nonfunctional, out-of-frame, *GFP* gene and allow the associated green fluorescence phenotype.

### Confirmation Of Cas9/sgrna-Directed Mutagenesis Using Dna Sequencing

To confirm Cas9/sgRNA-directed mutagenesis of nonfunctional *GFP* genes and to determine the nature of the mutagenesis, we conducted a separate experiment in which we sequenced five independent clones of PCR products amplified from target gene DNAs isolated from each of five different T1 plants (#21–#25), three of which were GFP positive and two of which were GFP negative. Prior to PCR amplification, DNA isolated from each plant had been treated with *Apa*LI to prevent amplification of nonmutagenized target region DNAs (i.e., those containing an intact *Apa*LI restriction site) and to enrich for DNA regions carrying target sites with altered *Apa*LI recognition sequences.

**Figure 6:**
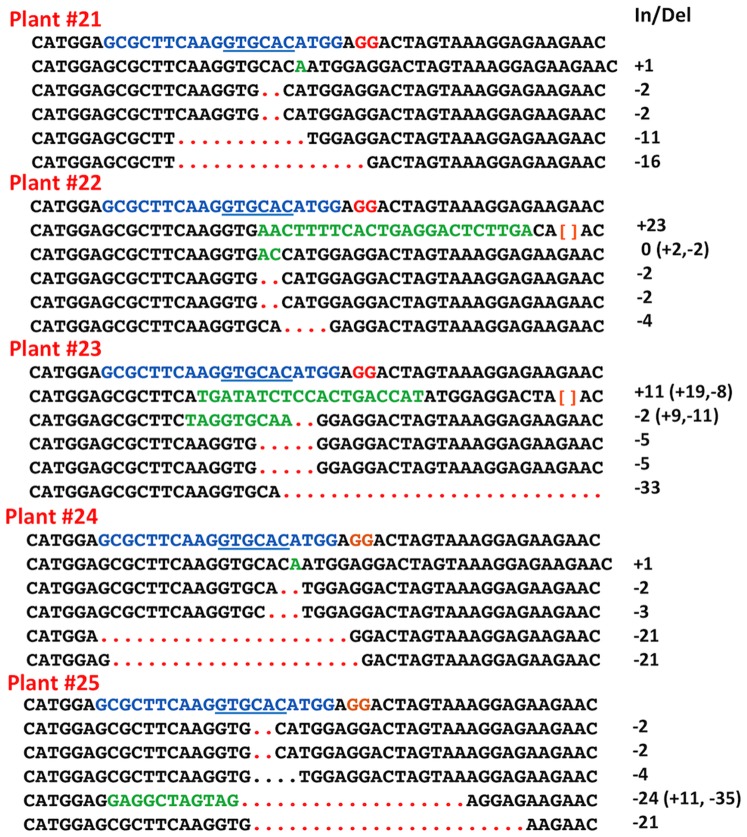
Confirmation by DNA sequencing of Cas9/sgRNA-mediated mutagenesis of the sgRNA target site within the nonfuctional, out-of-frame, *GFP* gene. Five cloned DNA fragments of 250 bp containing DNA from PCR amplified sgRNA target regions of (previously) nonfunctional *GFP* genes from three different GFP-positive T1 Arabidopsis plants (Plants 21, 22 and 25) and two different GFP-negative plants (Plants 23 and 24) were subjected to DNA sequencing. DNA sequences of a segment of 48 nucleotides surrounding the sgRNA target site are shown for each clone with the sequence of the nonmutagenized DNA region shown as the top line of each group. The 20 nucleotide target sequence for the Cas9/sgRNA complex is depicted in blue, the PAM site in red and the *Apa*LI recognition site is underlined in blue. Brackets ([]) denote nondisplayed DNA sequences. For the Cas9/sgRNA-mutagenized DNA sequences, deleted nucleotides are depicted as red dots and inserted nucleotides are shown in green. The net length of insertions and/or deletions (In/Del) are presented in the column to the right.

All five T1 plants, whether GFP-positive or GFP-negative, contained multiple PCR products displaying site-specific nucleotide insertions and deletions - hallmarks of Cas9/sgRNA-catalyzed DNA cleavage and NHEJ DNA repair (Figure 6). As expected for DNA repair events performed by the NHEJ system, most of the repairs involved insertion or deletions of only a few nucleotides, but with occasional creation of sizable deletions of 20 or more nucleotides. Also as expected for Cas9/sgRNA DNA cleavages that occur two or three base pairs upstream of the PAM site, all of the observed insertions/deletions were located in close proximity to the expected cleavage site.

The DNA sequence analyses shown in Figure 6 demonstrate the presence of multiple different mutagenized *GFP* target sequences within single leaves from five T1 Arabidopsis plants. These observations suggest that independent cas9/sgRNA-driven mutagenesis events occurred in somatic cells during seed or seedling development. This conjecture is consistent with the observation of mosaic patterns of GFP expression in T1 Arabidopsis plant leaves (Figure 3) in which some patches of cells display green fluorescence and neighboring patches do not. Together, these observations strongly suggest that even though Cas9 and sgRNA genes possibly become active soon after insertion of T-DNA into a host chromosome following floral dip transformation of Arabidopsis, the action of the Cas9/sgRNA complex in targeting the 20 bp sequence in the nonfunctional *GFP* gene is often not immediate, but occurs perhaps more or less randomly in cells during portions of seed or seedling development. These observations also demonstrate that the Cas9/sgRNA complex acted to modify nonfunctional *GFP* genes that were part of a T-DNA region stably integrated into a chromosomal location. Thus, the findings of the present study in regard to inheritance of *GFP* genes should also pertain equally to inheritance of endogenous Arabidopsis genes modified by the Cas9/sgRNA complex.

### Evidence For Inheritance Of Cas9/sgrna Derived Mutant Phenotypes And Genotypes In T2 Plants

All 24 transgenic T1 plants examined for this portion of our studies produced normal levels of seeds with generally high levels of viability. That is, there was no evidence for an effect of Cas9 and sgRNA gene expression on the ability of T1 plants to produce viable progeny. Indeed, examination of the Arabidopsis genome for sequences with sufficient homology to the 20 bp sgRNA target sequence to cause potential off-site targeting [e.g., 26, 30, 31] failed to reveal any sites of concern [19].

Germination and seedling survival rates of 70% to 80% in the presence of hygromycin were obtained for T2 generation seeds from the individual T1 plants tested suggesting that, in most cases, the hygromycin resistance gene (and accompanying Cas9, sgRNA and *GFP* genes) was present in a single copy in T2 plants. Leaves from T2 seedlings derived from 6 different T1 plants (T1 Plants #3 to #8) were excised and examined for green fluorescence using a confocal fluorescence microscope. T2 seedlings from all but one of the 6 T1 progenitor plants displayed no green fluorescence. All of the T2 seedlings derived from T1 Plant #6 had leaves displaying uniform green fluorescence (Figure S2).

**Figure 7:**
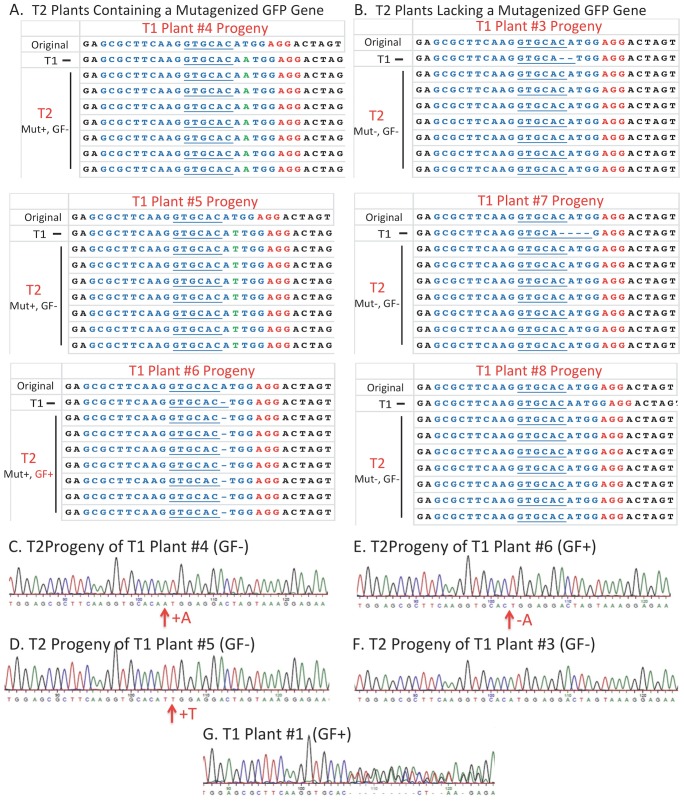
Confirmation of inheritance of a single modified or nonmodified *GFP* gene in each of 7 T2 progeny from 6 individual T1 generation plants – and evidence of Cas9 gene and/or sgRNA gene silencing in T2 progeny. DNA was isolated from each of 7 T2 progeny from each of 6 different progenitor T1 plants (T1 Plants #3 to #8). PCR was used to amplify a 250 bp DNA fragment containing the sgRNA target region of the nonfunctional, out-of-frame, *GFP* gene. DNA sequencing of the fragment provided the sequence of the 31 bp region displayed for each of the 42 T2 plants. The DNA sequence of the original *GFP* gene is provide as the top line in each column along with the sequence of one mutagenized *GFP* gene found in a leaf of the original progenitor T1 plant. A) DNA sequences of three groups of T2 plants in which there has been a Cas9/sgRNA-mediated gene modification including insertion of an A nucleotide (Plant #4 progeny), a T nucleotide (Plant #5 progeny), or deletion of an A nucleotide (Plant #6 progeny) that restored a proper reading frame and resulted in T2 progeny displaying a green fluorescence phenotype, B) DNA sequences of three groups of T2 plants (progeny of T1 Plants #3, #7 and #8) in which there was no inherited Cas9/sgRNA-mediated gene modification. (GF−), No green fluorescence phenotype; (GF+), Green fluorescence phenotype; (Mut+), Inherited mutagenized *GFP* gene; (Mut-), No inherited mutagenized *GFP* gene. C, D, E, and F) DNA sequencing traces from sequencing of PCR amplified Cas9/sgRNA target sites from a single leaf of an individual T2 progeny from T1 progenitor Plants #3, #7 and #8, respectively. G) A DNA sequencing trace from sequencing of the PCR amplified Cas9/sgRNA target sites isolated from a single leaf of T1 Plant #1 showing multiple overlapping DNA peaks caused by the presence of multiple different DNA sequences in the separate mutagenized *GFP* genes present in different patches of cells scattered throughout the leaf.

To determine if only a single gene was inherited in the T2 progeny of the 6 T1 plants examined above (T1 Plants #3 to #8), DNA was extracted from 7 hygromycin-resistant T2 plants derived from each of the 6 T1 progenitors. The 250 bp region containing the target site for Cas9/sgRNA cleavage was PCR amplified from each DNA preparation and subjected (without cloning) to DNA sequence analysis. Only one DNA sequence was obtained for each of the 7 individual T2 plants in each of the 6 groups (Figure 7A and B). For T2 plants derived from T1 generation plants #4, #5 and #6, the presence of a single mutagenized GFP gene sequence provided compelling evidence that, in each case, only a single *GFP* gene had been inherited from the T1 progenitor plant. For T2 plants derived from T1 plants #3, #7, and #8, the gene number could not be determined because only the original *GFP* gene sequence found in the transforming T-DNA were present. The sequencing data for T2 progeny of T1 Plant #6 revealed restoration of a proper reading frame in the *GFP* gene in these plants due to deletion (in the genetically competent cells of the T1 progenitor plant) of a single A nucleotide and the consequential expression of the green fluorescence phenotype noted above (Figure S2). The presence of only a single *GFP* gene in each of the T2 plants was further confirmed by careful examination of the DNA sequencing trace from one individual plant from each of the 6 groups of T2 plants. In all 6 cases (in which *GFP* gene was mutagenized or not) this examination showed uniform distribution of peaks with no indication of minor overlapping peaks (Figure 7C through F). As an example of a pattern that might be expected if more than one species of Cas9/sgRNA-altered *GFP* gene was present (or if a copy of the original nonfunctional *GFP* gene and a copy of a Cas9/sgRNA-altered *GFP* gene were both present), Figure 7G displays a DNA sequencing trace of the same region after PCR amplification of DNA isolated from leaves of T1 Plant #1– a plant that was shown earlier to contain multiple altered and nonaltered *GFP* genes by *Apa*LI restriction enzyme digestion of PCR amplified *GFP* genes (Figure 5). In this case, the uniform pattern of DNA sequence peaks upstream of the sgRNA target site becomes overlapping and lower in height as the sequencing trace proceeds through the area of the genes containing deletions and insertions caused by Cas9/sgRNA-mediated DNA cleavage and NHEJ DNA repair. From this set of experiments, we conclude that when coupled with the segregation ratios of hygromycin-resistant to hygromycin-sensitive T2 seedling (∼3∶1), the DNA sequence data provided in Figure 7 for T2 progeny of T1 generation plants #4, #5 and #6 provide compelling evidence for inheritance of only single *GFP* genes in the T2 progeny of each of the three different T1 progenitors – implying the presence of only a single T-DNA region in the genetically-competent cells of T1 plants #4, #5 and #6 used to produce the T2 and T3 generation plants analyzed in this study.

The data of Figure 7 also revealed a potentially important and unexpected finding – the probable silencing of the Cas9 gene and/or the sgRNA gene in progeny of T1 plants. Remembering the large amount of DNA sequencing information described earlier (Figure 6) that showed multiple different mutagenized *GFP* genes in single leaves from individual T1 plants, it is clear that the Cas9 and sgRNA genes were active in many different cells during T1 seed development (and, potentially, leaf development). Thus, the lack of mutagenesis of *GFP* gene sequences observed in leaves of three different groups of T2 plants (those produced from T1 plants #3, #7 and #8, Figure 7) and the lack of expression of a functional GFP in any leaf of these plants suggest that the Cas9 and/or sgRNA genes in these T2 plants may have been silenced in the genetically competent cells of progenitor T1 plants or during early T2 seed development. Confirmation of silencing and its timing relative to seed and plant development will require future studies. Because of the possibility that Cas9 and sgRNA gene silencing can occur even during early development of T1 generation seeds and is likely not uniform in timing between different cells and tissues during seed and/or leaf development, attempts to accurately define the timing of gene silencing likely will be difficult. That is because such studies may require highly sensitive RT-PCR or RNA-seq procedures to detect the presence or absence of Cas9 and sgRNA transcripts from minute samples of various tissues taken during the entire course of development of multiple T1 generation Arabidopsis seeds, seedlings and plants – a challenging, but potentially revealing, task.

As an extension of our experiments showing stable inheritance of genes modified by the Cas9/sgRNA system in the T2 generation, we extracted DNA from several T3 generation progeny of T2 plants representing the three sets of T2 plants containing mutagenized GFP gene sequences examined in Figure 7. As expected, all plants displayed a single mutagenized GFP gene sequence that was identical to the one found in the T2 plant from which they were derived (Figure S3) - providing verification of stable inheritance of the mutagenized GFP genes in the T3 generation. A summary of the inheritance data from the present study is provided in Table S1.

Together, results of the present study point to the ease and high efficiency in producing T1 Arabidopsis plants that successfully express the Cas9/sgRNA system for targeted gene modification. These studies also verify stable inheritance of newly acquired genes/traits in T2 and T3 plants. The unexpected discovery of an apparent lack of Cas9/sgRNA activity in T2 and T3 plants suggests a potential role for gene silencing in dictating the extent and duration of Cas9/sgRNA-mediated mutagenesis during plant development.

An important virtue of the Cas9/sgRNA system for plant biotechnology applications is that once a desired gene modification has been achieved, the T-DNA region carrying Cas9, sgRNA and other transgenes can be eliminated entirely from progeny by simple genetic crosses – as we have demonstrated earlier for TALEN-modified rice plants (3). The lack of transgenes may allow such genetically enhanced crop plants to avoid present regulatory restraints and, thus, make their way into the marketplace faster than is presently the case for transgenic plants. Presuming that future studies demonstrate activity of the Cas9/sgRNA system in a wide range of plants, this system for precise and efficient gene modification would appear to have significant promise for greatly speeding scientific advances in plant biology and for engineering crop plants with improved traits for productivity and nutrition.

After this article was submitted for publication and distributed for review, a manuscript by Feng et al. [32] appeared that reported inheritance of Cas9/sgRNA-generated mutant genes in T2 generation Arabidopsis plants and other results that support the major conclusions of the present article.

## Materials And Methods

### Preparation Of Plant Materials

*Arabidopsis thaliana* ecotype (Col-0) was used in all experiments. Seeds were sown on soil and were stratified for 3 days at 4°C. Seeds were geminated and plants were grown within a controlled environment chamber at 22°C with a 12 hr light/12 hr dark photoperiod and under 75% relative humidity till early flowering stage about 4 weeks from germination. For selection of transgenic Arabidopsis that are resistant to antibiotic hygromycin, seeds were surface sterilized by exposure to 100% ethanol for one minute and then by treatment with 50% commercial bleach for 5 minutes. After three thorough washes with water, the seeds were sown on 1% agarose plates containing MS medium with a final concentration of 20 µg mL^−1^ hygromycin and 100 µg mL^−1^ carbenicillin. Plates containing the seeds were incubated in the controlled growth chamber described above. Germinated seedlings with resistance to hygromycin were transferred to soil ten days after germination.

### Construction Of Plant Expression Vectors

Design and construction of the Cas9 gene and sgRNA gene from *Streptococcus pyogenes* [6],[9], the nonfunctional, out-of-frame *GFP* gene, and the *Agrobacterium tumefaciens* binary vector containing these gene constructs have been described earlier [16]. A map of the binary vector used in the present studies as well as the complete DNA sequence of the T-DNA region of the binary vector are provided as Figures S3 and S4, respectively.

### Floral Dip-*Agrobacterium*-Mediated Arabidopsis Transformation

The transformation of Arabidopsis was performed as described earlier [27]. In brief, *Agrobacterium tumefaciens*, strain C58cc, carrying the Cas9 gene, sgRNA gene, mutant *GFP* gene and a hygromycin resistance gene was grown overnight at 27°C with shaking at 200 RPM in 5 mL of Luria–Bertani (LB) medium supplemented with appropriate antibiotics. The next afternoon, 1.5 mL of the saturated bacterial culture was transferred to 500 mL of LB medium containing appropriate antibiotics and grown with shaking overnight at 27°C. Cells were harvested the next day by centrifugation and diluted in 5% sucrose buffer to a final OD_600_ of 0.8. Silwet L-77 was added to a final concentration of 0.01%. Inflorescence clusters of Arabidopsis plants were dipped into the bacterial culture [27]. Inoculated plants were maintained in growth chambers until seeds were fully mature. T1 generation seeds were collected from individual T0 plants and germinated on plates containing solid MS medium with 20 µg/mL hygromycin to select successfully transformed T1 plants and to determine rates of transformation obtained in the floral dip transformation protocol.

### Enrichment Of Mutagenized Target Sites Using Restriction Enzyme Digestion Of Pcr Amplified Sgrna Target Site Regions Present In Mutagenized *Gfp* Genes

PCR amplification of the sgRNA target sites in DNA from GFP-positive T1 Arabidopsis plants and subsequent restriction enzyme digestion were used to verify Cas9/sgRNA-stimulated cleavage and subsequent erroneous DNA repair by NHEJ. DNA was extracted from young T1 Arabidopsis leaves using a NucleoSpin Plant II kit (MACHEREY-NAGEL GmbH and Co.KG, Germany). To eliminate PCR amplification of most DNA sequences from the original nonfunctional *GFP* gene, the extracted DNA was cleaved at the resident *Apa*LI cut site that is located within the 20 bp sgRNA target site near the PAM site. In this way, subsequent PCR amplification favored production of mutagenized DNA. For PCR amplification, primers 125 bp upstream and 125 bp downstream of the *Apa*LI restriction site were employed. PCR amplified and *Apa*LI-digested DNA was agarose gel-purified and the resulting ∼250 bp DNA fragments were cloned into a pBlueScript vector. DNA sequencing of the 250 bp fragments was used to determine the types of Cas9/sgRNA/NHEJ-mediated mutations obtained.

### Dna Sequence Analysis Of Sgrna Target Regions Of 42 Individual T2 Plants

Total DNA was isolated from an individual leaf of each of 7 T2 progeny from 6 different T1 progenitor plants (i.e., 42 separate leaf samples). The same primers as described above were used to PCR amplify the ∼250 bp DNA region containing the sgRNA target site in DNA from each leaf. This PCR amplified DNA was sequenced directly (i.e., no cloning of fragments was involved) to determine if one or more than one modified or nonmodified *GFP* gene was present.

### Fluorescence Confocal Microscopy Of Arabidopsis Leaf Cells

Second or third true leaves of T1 and T2 seedlings were cut and analyzed for GFP fluorescence using a Nikon ECLIPSE 90i system confocal fluorescence microscope at 100× and 600× magnification. The excitation and detection wavelengths were set at 448 nm and 500–550 nm, respectively, for GFP fluorescence and at 641 nm and 662–737 nm for chlorophyll auto-fluorescence to minimize cross talk between the two fluorescence channels.

## Supporting Information

Figure S1 **Analyses of mutagenized sgRNA target sites in nonfunctional** ***GFP*** **genes by PCR/RE in DNAs from 24 T1 transgenic Arabidopsis plants.**
(PDF)

Figure S2 **Expression of a functional** ***GFP*** **gene in a leaf of a T2 progeny of T1 generation Plant #6.**
(PDF)

Figure S3 **Confirmation of inheritance of a single modified** ***GFP*** **gene in each of 7 T3 progeny from 3 individual T2 generation plants.**
(DOCX)

Figure S4 **Map of plant expression vector containing Cas9, sgRNA and mutant** ***GFP*** **genes.**
(DOCX)

Figure S5 **Sequences of plant expression vector containing Cas9, sgRNA and mutant** ***GFP*** **genes from LB to RB.**
(DOCX)

Table S1 **Summary of expression of Cas9/sgRNA-induced mutations of the targeted nonfunctional GFP gene in somatic tissue of T1 generation plants and inheritance of the mutagenized GFP gene in T2 and T3 generation Arabidopsis plants.**
(DOCX)
