# Supplementary material for: Efficient CRISPR/Cas9-Mediated Gene Editing in Arabidopsis thaliana and Inheritance of Modified Genes in the T2 and T3 Generations
Source: PLoS One. 2014 Jun 11;9(6):e99225. doi: 10.1371/journal.pone.0099225 (PMC4053344; doi:10.1371/journal.pone.0099225)
Supplement: Figure S1 — (PDF) [file pone.0099225.s001.pdf]

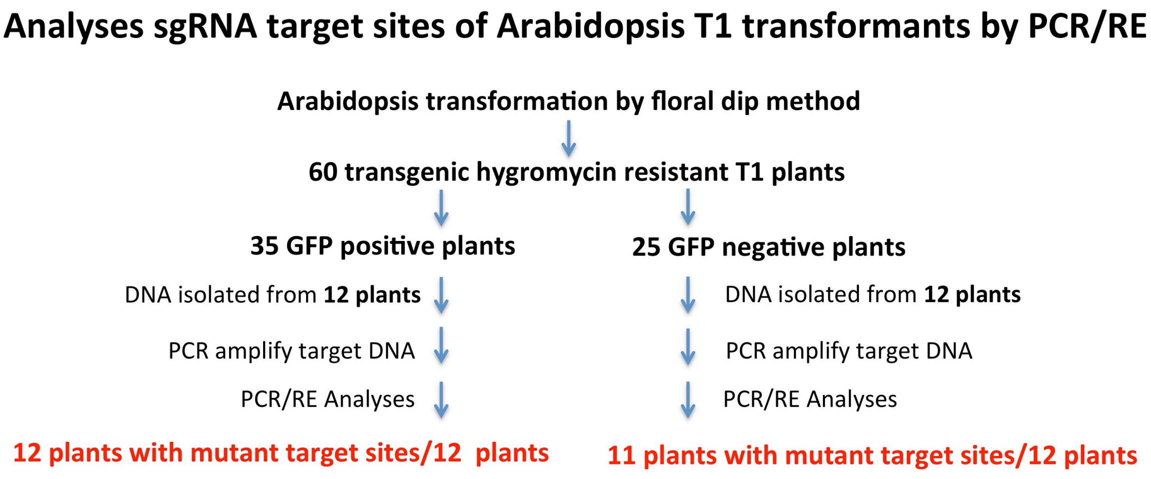

Figure S1. Analyses of mutagenized sgRNA target sites in nonfunctional *GFP* genes by PCR/Restriction Enzyme (PCR/RE) in DNAs from 24 T1 transgenic Arabidopsis plants.
