# Supplementary material for: Efficient CRISPR/Cas9-Mediated Gene Editing in Arabidopsis thaliana and Inheritance of Modified Genes in the T2 and T3 Generations
Source: PLoS One. 2014 Jun 11;9(6):e99225. doi: 10.1371/journal.pone.0099225 (PMC4053344; doi:10.1371/journal.pone.0099225)
Supplement: Figure S2 — (PDF) [file pone.0099225.s002.pdf]

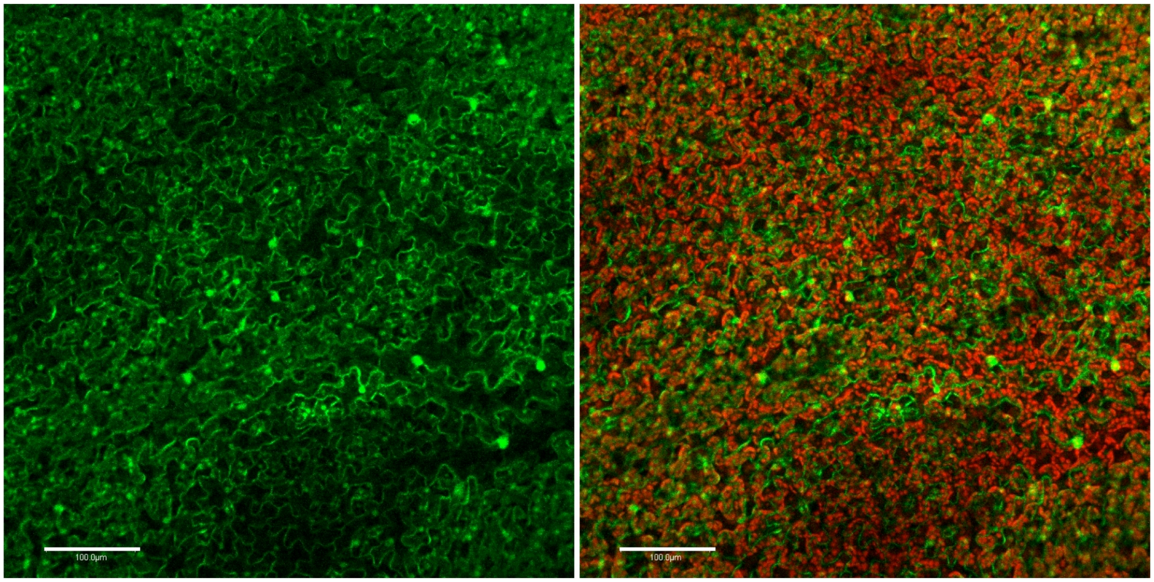

Figure S2. Expression of a functional *GFP* gene in a leaf of a T2 progeny of T1 generation Plant #6. A) Detection of green fluorescence protein signals in a transgenic leaf. B) Merged image of red chlorophyll fluorescence and GFP fluorescence. Bar, 100 µm.
