# Supplementary material for: Efficient CRISPR/Cas9-Mediated Gene Editing in Arabidopsis thaliana and Inheritance of Modified Genes in the T2 and T3 Generations
Source: PLoS One. 2014 Jun 11;9(6):e99225. doi: 10.1371/journal.pone.0099225 (PMC4053344; doi:10.1371/journal.pone.0099225)
Supplement: Figure S3 — (DOCX) [file pone.0099225.s003.docx]

**
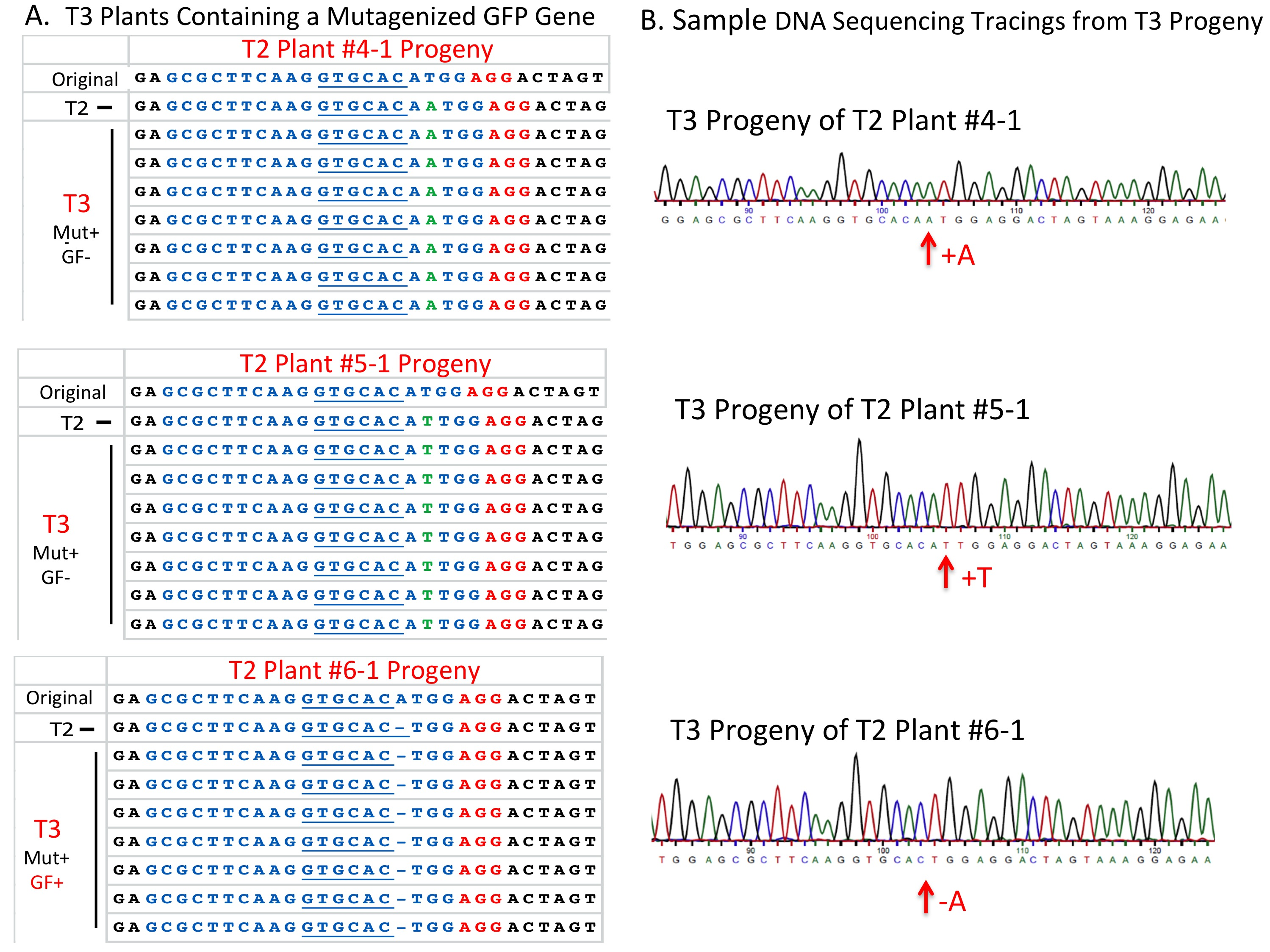
**

**Figure S3.** Confirmation of inheritance of a single modified *GFP* gene in each of 7 T3 progeny from 3 individual T2 generation plants. DNA was isolated from each of 7 T3 progeny from each of 3 different progenitor T2 plants (i.e., T2 Plants #4-1, #5-1 and #6-1 derived from T1 Plants #4, #5 and #6, respectively). PCR was used to amplify a 250 bp DNA fragment corresponding to the sgRNA target region of the original nonfunctional, out-of-frame, *GFP* gene. DNA sequencing of the fragment provided the sequence of the 31 bp region displayed for each of the 21 T2 plants (7 plants derived from T2 Plant #4-1, 7 plants derived from T2 Plant #5-1, and 7 plants derived from T2 Plant #6-1). The DNA sequence of the original *GFP* gene is provide as the top line in each column followed in the second line with the sequence of the single mutagenized *GFP* gene found in the progenitor T2 plant. A) DNA sequences in each of three groups of T3 plants are identical to those of their T2 predecessor (Figure 7) in which there had been a Cas9/sgRNA-mediated gene modification [i.e., insertion of an A nucleotide (Plant #4-1 progeny), a T nucleotide (Plant #5-1 progeny), or deletion of an A nucleotide (Plant #6-1 progeny)]. (GF-), No green fluorescence phenotype; (GF+), Green fluorescence phenotype; (Mut+), Inherited mutagenized *GFP* gene. B) DNA sequence traces of PCR amplified GFP target sites from one representative plant from each group of three T3 progeny.
