# Supplementary material for: Efficient CRISPR/Cas9-Mediated Gene Editing in Arabidopsis thaliana and Inheritance of Modified Genes in the T2 and T3 Generations
Source: PLoS One. 2014 Jun 11;9(6):e99225. doi: 10.1371/journal.pone.0099225 (PMC4053344; doi:10.1371/journal.pone.0099225)
Supplement: Figure S4 — (DOCX) [file pone.0099225.s004.docx]

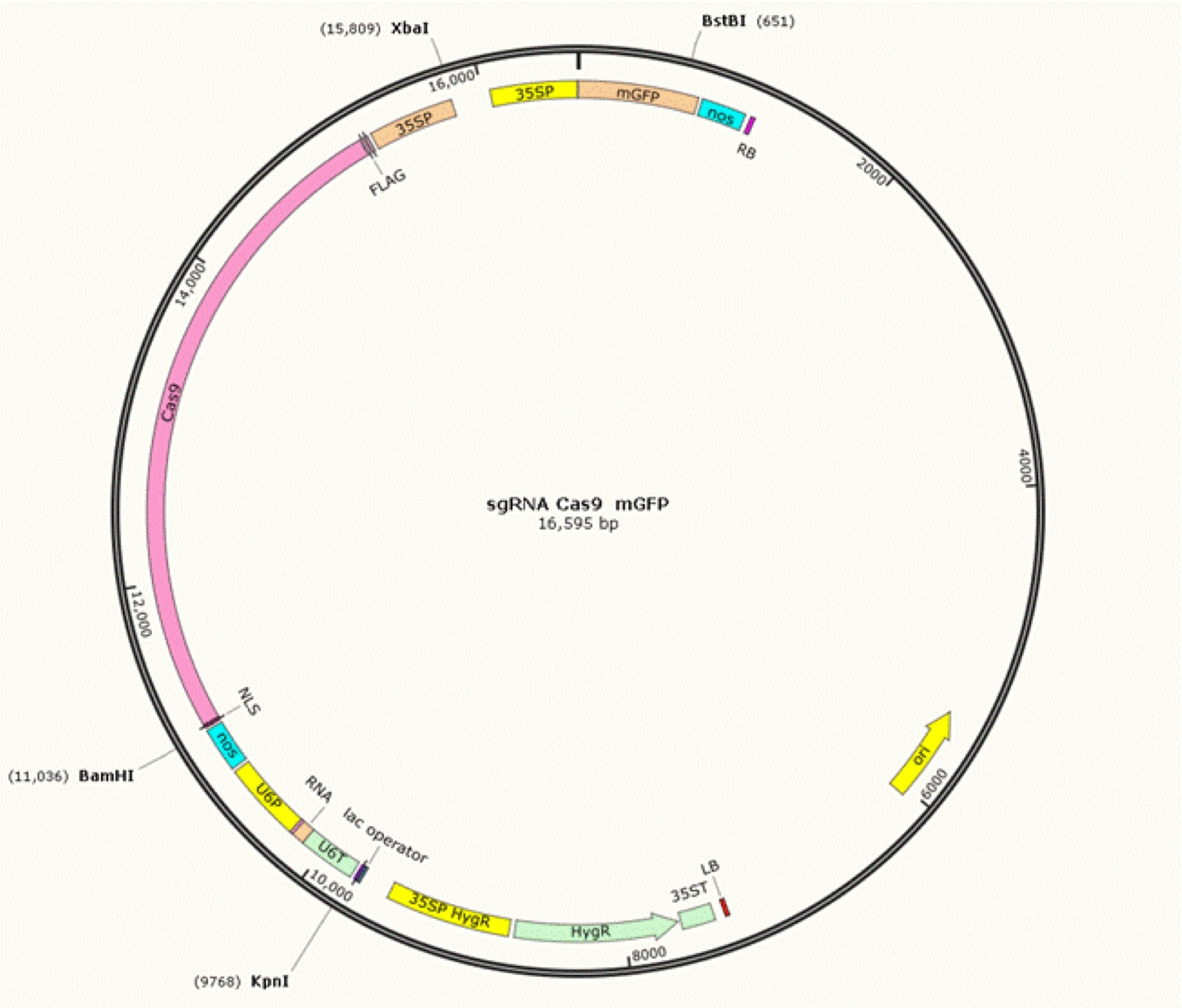


**Figure S4**. Map binary vector containing Cas9, sgRNA, nonfunctional *GFP* genes and hygromycin resistance genes.
