# Supplementary material for: Efficient CRISPR/Cas9-Mediated Gene Editing in Arabidopsis thaliana and Inheritance of Modified Genes in the T2 and T3 Generations
Source: PLoS One. 2014 Jun 11;9(6):e99225. doi: 10.1371/journal.pone.0099225 (PMC4053344; doi:10.1371/journal.pone.0099225)
Supplement: Table S1 — (DOCX) [file pone.0099225.s006.docx]

**Table S1.** Summary of expression of Cas9/sgRNA-induced mutations of the targeted nonfunctional GFP gene in somatic tissue of T1 generation plants and inheritance of the mutagenized GFP gene in T2 and T3 generation Arabidopsis plants.

1. **Efficiency of Cas9/sgRNA-induced GFP Target Gene Mutagenesis in Somatic Cells of T1 Arabidopsis Leaves**

| Plant Number | 1 | 2 | 3 | 4 | 5 | 6 | 7 | 8 | 9 | 10 | 11 | 12 | **Ave** | Con |
| --- | --- | --- | --- | --- | --- | --- | --- | --- | --- | --- | --- | --- | --- | --- |
| % Mod. GFP Genes | 37 | 52 | 44 | 28 | 56 | >95 | 51 | 25 | 76 | 83 | 51 | 55 | **~54** | 0 |

Data from Figure 5

1. **Inheritance of Modified and Nonmodified Target GFP Genes in T2 Plants (Verified by DNA Sequencing Data)**

| T1 Plant Number | #3 | #4 | #5 | #6 | #7 | #8 | Mutagenized GFP Gene Inheritance |
| --- | --- | --- | --- | --- | --- | --- | --- |
| Mutagenized GFP Gene  In T2 Gen. | No | Yes | Yes | Yes | No | No | 3/6 T2 Plants  (~50%) |
| # GFP Genes Present | * | 1 | 1 | 1 | * | * |  |
| GFP Gene Functional | No | No | No | Yes | No | No |  |

Data from Figure 7 *At least one nonmutagenized GFP gene present

1. **Inheritance of Modified and Nonmodified Target GFP Genes in T3 Plants (Verified by DNA Sequencing Data)**

| T1 Plant Number | #4 | #5 | #6 | Mutagenized GFP Gene Inheritance |
| --- | --- | --- | --- | --- |
| Mutagenized GFP Gene  In T2 Gen. | Yes | Yes | Yes | 3/3 T3 Plants  (100%) |
| # GFP Genes Present | 1 | 1 | 1 |  |
| GFP Gene Functional | No | No | Yes |  |

Data from Supplementary Materials Figure S3
